# Myc Supports Self-Renewal of Basal Cells in the Esophageal Epithelium

**DOI:** 10.3389/fcell.2022.786031

**Published:** 2022-03-04

**Authors:** Tomoaki Hishida, Eric Vazquez-Ferrer, Yuriko Hishida-Nozaki, Yuto Takemoto, Fumiyuki Hatanaka, Kei Yoshida, Javier Prieto, Sanjeeb Kumar Sahu, Yuta Takahashi, Pradeep Reddy, David D. O’Keefe, Concepcion Rodriguez Esteban, Paul S. Knoepfler, Estrella Nuñez Delicado, Antoni Castells, Josep M. Campistol, Ryuji Kato, Hiroshi Nakagawa, Juan Carlos Izpisua Belmonte

**Affiliations:** ^1^ Gene Expression Laboratory, Salk Institute for Biological Studies, La Jolla, CA, United States; ^2^ Laboratory of Biological Chemistry, School of Pharmaceutical Sciences, Wakayama Medical University, Wakayama, Japan; ^3^ Department of Basic Medical Sciences, Graduate School of Pharmaceutical Sciences, Nagoya University, Nagoya, Japan; ^4^ Department of Cell Biology and Human Anatomy, University of California, Davis, Davis, CA, United States; ^5^ Universidad Católica San Antonio de Murcia (UCAM), Campus de los Jerónimos, Murcia, Spain; ^6^ Gastroenterology Department, Hospital Clinic, CIBEREHD, IDIBAPS, University of Barcelona, Barcelona, Spain; ^7^ Division of Gastroenterology, Department of Medicine, Perelman School of Medicine, Philadelphia, PA, United States; ^8^ Abramson Cancer Center, University of Pennsylvania, Philadelphia, PA, United States

**Keywords:** MYC, cancer, senescence, aging, mitochondria highlights

## Abstract

It is widely believed that cellular senescence plays a critical role in both aging and cancer, and that senescence is a fundamental, permanent growth arrest that somatic cells cannot avoid. Here we show that Myc plays an important role in self-renewal of esophageal epithelial cells, contributing to their resistance to cellular senescence. Myc is homogeneously expressed in basal cells of the esophageal epithelium and Myc positively regulates their self-renewal by maintaining their undifferentiated state. Indeed, Myc knockout induced a loss of the undifferentiated state of esophageal epithelial cells resulting in cellular senescence while forced MYC expression promoted oncogenic cell proliferation. A superoxide scavenger counteracted Myc knockout-induced senescence, therefore suggesting that a mitochondrial superoxide takes part in inducing senescence. Taken together, these analyses reveal extremely low levels of cellular senescence and senescence-associated phenotypes in the esophageal epithelium, as well as a critical role for Myc in self-renewal of basal cells in this organ. This provides new avenues for studying and understanding the links between stemness and resistance to cellular senescence.

## Highlights

• Esophageal epithelia show resistance to senescence in mice.
• c-Myc is homogeneously expressed in basal cells of the esophageal epithelium.
• Myc is required for stemness-associated inhibition of senescent characteristics in basal cells of the esophageal epithelium.
• Basal cells of the esophageal epithelium have low levels of mitochondrial activity.

## Introduction

Most cells do not proliferate indefinitely, but instead enter cellular senescence, a permanent cell cycle arrest triggered by excessive rounds of cell division, oncogenic stimuli, or genotoxic stresses (Kuilman et al., 2010). Senescence is thought to be a fundamental feature of somatic cells, and is known to contribute to organismal aging and prevention of cancer initiation (Hanahan, 2022). However, there are cell-type specific differences in the induction and effectiveness of senescence (Lopez-Otin et al., 2013). For example, rodent glia cells (rat Schwann cells) do not exhibit replicative senescence *in vitro* (Mathon et al., 2001), nor do cultured epidermal stem cells that have been derived from the footpad (Stern and Bickenbach, 2007; Doles et al., 2012). Thus, some cell types, such as adult/tissue stem cells and progenitors, show resistance to senescence. However, it is unknown whether there is tissue *in vivo* that can similarly evade senescence.

Here we found that the esophagus did not exhibit aging features in mice. Forced-expression of MYC induced oncogenic cell proliferation while MYC knockout reduced the self-renewal capacity of esophageal epithelial cells, which resulted in cellular senescence, indicating the importance of MYC in preserving their self-renewal. It was also suggested that MYC is necessary for proliferating cells to keep an undifferentiated state and maintain a low level of mitochondrial superoxide. Taken together, these data revealed an essential role of MYC on the stemness of esophageal epithelial cells, which are highly resistant to senescence.

## Materials and Methods

### Mice

*Myc* ^Myc-GFP/Myc-GFP^ (Huang et al., 2008), *Sox2* ^CreER/WT^ (Arnold et al., 2011), *Sox2* ^GFP/WT^ (Arnold et al., 2011), *ROSA* ^LSL-GFP/LSL-GFP^ (Mao et al., 2001), tetO-*MYC* (Felsher and Bishop, 1999) and *ROSA* ^LSL-rtTA-IRES-GFP/LSL-rtTA-IRES-GFP^ (Belteki et al., 2005) have been previously described. LMNA^G609G^ mice were generated by Carlos López-Otín at the University of Oviedo, Spain and kindly donated by Brian Kennedy at the Buck Institute. We generated Myc cdKO mice from Myc cdKO ESCs (Varlakhanova et al., 2010). Genotyping was performed by using the primer set which are suggested to be used in The Jackson Laboratory. Genotyping for Myc was performed as described previously (Varlakhanova et al., 2010). We used both male and female mice for this study but the same gender was used for each experiment unless otherwise stated. To activate Cre in the mice carrying CreER, TAM, dissolved in corn oil, was given orally (50 mg/ml) to 3- to 12-week-old animals for 3 consecutive days, if not otherwise stated. Dox was administered in drinking water (0.5 mg/ml), starting with TAM treatment. All animal experiments were approved by the Salk Institute for Biological Studies IACUC and conform to regulatory standards.

### Ki67 Immunostaining and Cell Quantification

Esophagi and small intestines were dissected from young, old and LMNA G609G mice and washed with PBS, followed by fixation with 4% paraformaldehyde for 24 h at 4°C. The tissues were then soaked in 15% sucrose in PBS for at least 12 h and 30% sucrose in PBS for 24 h before being embedded in optimal cutting temperature (OCT) (Tissue-Tek) prior to cryo-sectioning. The prepared sections (8–10 μm) were washed twice in Tris-buffered saline (TBS, *pH* = 7.0) to remove OCT followed by antigen retrieval using HistoVT One (Nacalai tesque) according to manufacturer’s instructions. Sections were then blocked for 1–2 h in 6% normal horse serum in TBST (TBS + 0, 5% Triton X-100) and incubated overnight at 4°C with primary antibodies anti-Ki67 (Cell signaling, 12,202, 1:100). Alexa Fluor 488-conjugated donkey anti-Rabbit IgG (Molecular Probe, 1:200) was used as a secondary antibody and nuclei were stained with 4′6-diamidino-2-phenylindole (DAPI). Images were acquired using a Zeiss LSM 780 laser-scanning microscope (Carl Zeiss Jena) at 10×, 20×, and 63× magnification. For the quantification, several images were taken at 20× or 63× and underwent the stitching function of ImageJ (National Institute of Health) to reconstruct the whole tissue. At least, three sections per tissue from three animals were used for the analyses. Cells positive for each marker were counted in a blinded manner using ImageJ. For the esophagus, the percentage of Ki67^+^ cells was assessed on nine sections from three mice of each group (young, old and LMNA G609G mice). For the small intestine, the number of Ki67^+^ cells was counted from at least 16 crypts from three mice of each group.

### IHC and SA-βGal Staining

For IHC, tissues were harvested, fixed in 10% neutralized Formalin for 2 days and then stored in 70% ethanol until further processing. H&E staining, PAS staining and IHC on paraffin-section were performed following standard protocols. The following antibodies were used for IHC: anti-GFP (Abcam, 6673, 1:200; Clontech, JL-8, 1:100); Ki67 (Cell signaling, 12202, 1:200). SA-βgal staining was performed as previously described (Debacq-Chainiaux et al., 2009).

### RNA Isolation and Quantitative-PCR

**FIGURE 1:**
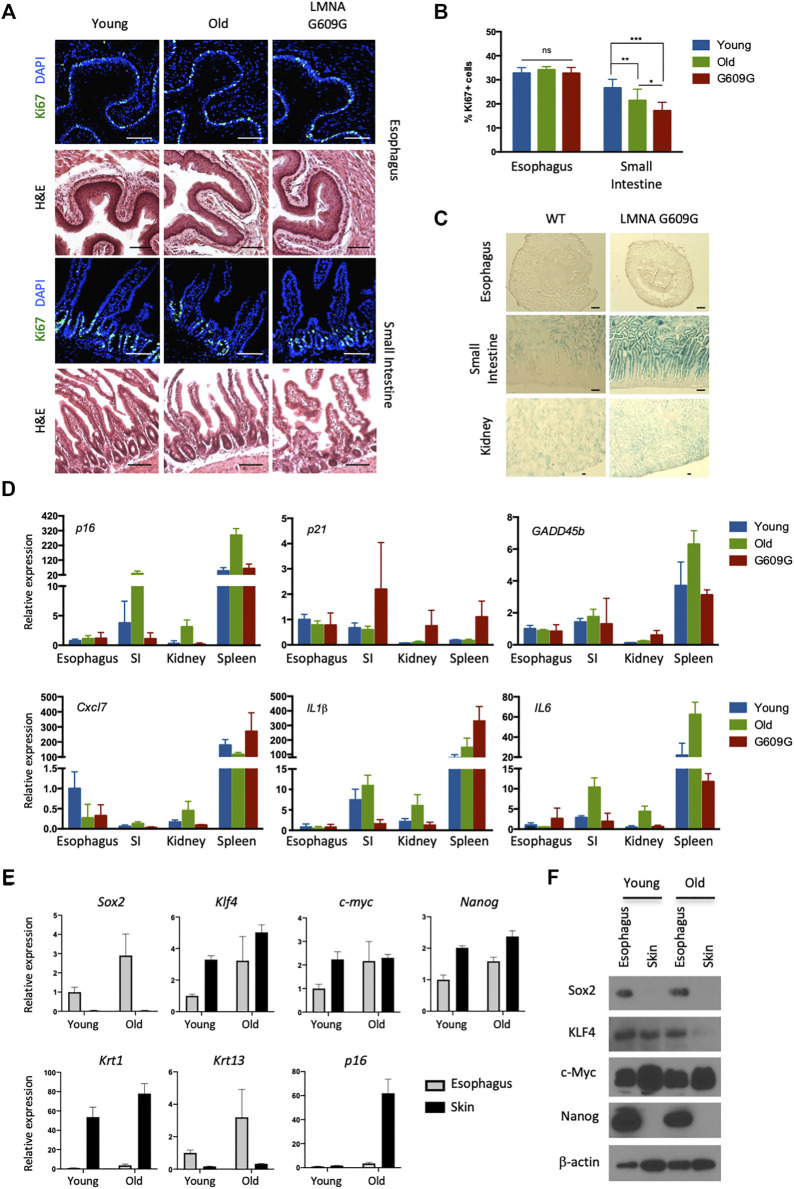
No visible senescence of esophageal epithelial cells expressing pluripotency factors. **(A)** Representative immunofluorescent pictures of Ki67 (green) staining and H&E staining in the esophagus and small intestine for young (3 months old), old (22 months old) and LMNA G609G HGPS mouse model (3 months old). Scale bars, 100 μm. **(B)** Proliferative index of the esophagus and small intestine. We quantified Ki67^+^ cells in the esophagus and small intestine from three mice for each group. Data represent the mean with SD. *ns* = non-significant, *p < 0.01, **p < 0.001, ***p < 0.0001. **(C)** SA-βgal staining in the esophagus, small intestine and kidney for both young (3 months old) and LMNA G609G HGPS mouse models (3 months old). Scale bars, 100 μm. **(D)** qPCR analysis for aging markers. SI: Small Intestine. Data represent the mean with SE (*n* = 3). **(E)** qPCR analysis for the esophagus and the skin from young and old mice. Krt1 and krt13 were used as specific keratins for the skin and the esophagus, respectively. Data represent the mean with SE (*n* = 3). **(F)** Western blotting for pluripotency factors. Epithelial cells were isolated from the indicated tissues and cultured for 1 week before cell lysate preparation.

Total RNAs were isolated using TRIzol reagent (Invitrogen) and RNeasy Mini kit (Qiagen) according to the manufacturer’s instructions. RNA samples were treated with RNase-Free DNase Set (Qiagen). RT was performed with SuperScript III (Invitrogen) followed by qPCR using Platinum SYBR Green quantitative PCR super mix (Invitrogen) in a thermocycler. The levels of expression of respective genes were normalized to corresponding GAPDH values or Nat1 values, and the normalized values were divided by those of the corresponding standard samples (Young Esophagus for Figures 1D,F; Untreated (-4OH) samples for Figure 4F; Control samples for Supplementary Figure S2). Primer sequences are listed in Supplementary Table S1.

### Esophageal Cell Culture

mEPCs were derived as previously described (Kalabis et al., 2008). Briefly, the esophagi were isolated, opened longitudinally, washed in PBS followed by Dispase (1 U/ml) for 15–20 min at 37°C. The opened esophagi were minced with forceps and incubated with trypsin for 10 min at 37°C. After inactivation of trypsin with FBS, the cell suspension was filtrated through 100-μm and 40-μm cell strainers. The obtained cells were centrifuged and re-suspended in SAGM (LONZA) containing 1 μM A-83-01, 1 μM DMH-1, 3 μM CHIR99021 and 10 μM Y-27632, followed by plating on matrigel-coated plates. To activate CreER or ER-Ras^V12^, the cells were treated with 0.1 μM 4OH. For cell cycle analysis, EdU-647 was used together with cell-cycle 405 according to manufacture’s protocol (ThermoFisher). Human primary esophageal epithelial cells were described in previous papers (Harada et al., 2003; Takaoka et al., 2004). 10058-F4 (Sigma) was used as a Myc inhibitor as needed.

### Plasmid Constructions and Viral Production

The ORF of ER-Ras^V12^ was amplified from pLNCX2-ER-Ras^V12^ (Addgene, #67844) and subcloned to pMX-IB with In-Fusion (Clontech). pMX-retroviral plasmids were transfected to PLAT-E cells using Lipofectamine 3000 (Invitrogen) according to manufacturer’s instructions. For tetO-MYC-2A-puro, the amplified ORF of MYC was subcloned to ptetO-2A-puro lentiviral vector, generated from pTetO-Ngn2-puro (Addgene, #52047). Viral supernatants were collected around 48 h after transfection and passed through a 0.45 μm filter to remove cellular debris and then the supernatants freshly prepared were incubated for 1 h while spinning at 800 × g, followed by changing to fresh medium. Two days after infection, the cells were selected with 20 μM blasticidin or 2 μg/ml puromycin for 6 days.

### Nanostring

Differential gene expression profiling was carried out with purified RNA using the Nanostring nCounter Pan Cancer Profiling Panel (Nanostring, Seattle, WA) according to manufacturer’s instructions.

### Live-Imaging and Image Analysis

Cell tracking experiments was performed using IncuCyte imaging system (Essen Bioscience). Images were automatically acquired from 6-well plates at ×10 magnification every 30 min for 80 h. Raw images were processed with two types of filter sets according to time period. For 0–40 h, the filter set 1 for non-confluent cells were applied as follows: (Step 1) Contrast enhancement by original source code in R (version 3.1.0) (R Development Core Team, https://www.r-project.org/). Pixel higher than 140 were converted into 255. (Step 2) Texture enhancement, (Step 3) Segmentation, (Step 4) Removal of small objects, and (Step 5) Fill holes (under area 20) were processed. Then recognized cellular objects were counted. During this period, individual cells were recognized sharply for cellular region. However, flat enlarged cells can only be recognized with their center nuclei area, and the cell recognition accuracy for their edge was not sharp. Therefore, during this period, only the total cell counts were used as measurement data. For the period after 40 h, the filter set 2 for confluent cells were applied as follows: From Step 1 to Step 3, the same processing was applied as filter set 1. (Step 4) Fill hole (under 20), (Step 5) Erode (2 pixels) were added to the processed images. Finally the recognized cellular objects were counted, labeled, and measured for their area size. The image processing was applied by CL-Quant (Nikon corp. Tokyo, Japan). To illustrate time-course growth, bar-whisker plots, and the size distribution in measured cells, original source code by R was applied.

For the detailed morphological analysis on four conditions (Control, +Ca, −ADCY, and −ADCY + Ca), the phase contrast images taken by phase contrast microscopy (OLYMPUS, IX51) were manually traced to measure the accurate morphology in both normal and flat enlarging cells. The detailed image processing is described in Supplementary Note S2.

For SA-βgal positive cell measurement, SA-βgal-stained color image and phalloidin-stained fluorescent image from the same FOV by fluorescent microscopy (OLYMPUS, IX51) were processed by CL-Quant. First, the SA-βgal stained images were converted into Blue image, and binalized (threshold > 100 intensity). From the binalized image, their stained area was constructed as a image mask. Second, the phalloidin stained fluorescent images were binalized (threshold 120), and remaining intensity = 96 pixels were converted by 255 to enhance the regional contrast per cells. Then the binalized images were constructed as the second image mask. These two image masks were merged, and recognized for cell labeling. In each cellular object, the SA-βgal stained area, covered by the first mask, were measured to calculate the SA-βgal positive area per cells. From the recognized cells, 300 cellular objects were randomly selected in the data processing, and used for their distribution analysis. All data analysis was done by original source code by R.

### Mitochondrial Analysis

Isolated, trypsinized cells were incubated with 100 nM TMRM (ThermoFisher) and 500 nM MitoSpy Green FM (BioLegend) at 37°C for 30 min in PBS containing 0.5% BSA and washed with PBS once, followed by FACS analysis.

### Statistic Analysis

For comparisons, unpaired *t* test or one-way ANOVA with Tukey’s *post hoc* analysis were used with GraphPad Prism 8 unless otherwise stated. Values with *p* < 0.05 are considered statistically significant.

## Results and Discussion

### A Lack of Senescence in the Esophagus

Recent deep- and micro-sequencing-based mapping of genetic mutations has revealed that normal esophageal epithelial cells exhibit age-dependent expansion of mutated clones, as well as higher levels of mutations than seen with sun-exposed skin cells. This suggests that esophageal epithelial cells robustly proliferate and survive long enough to accumulate many somatic mutations without cellular senescence (Martincorena et al., 2018; Yokoyama et al., 2019). We have also observed that basal esophageal epithelial cells continue to proliferate after exposure to oncogenic insults, namely Kras^G12D^ (Hishida et al., 2019) and PIK3CA^H1047R^ (data not shown). Based on these data, we speculated that esophageal epithelial cells may possess a unique ability to resist senescence. To address this hypothesis, we analyzed the proliferative capacity of esophageal cells in aged wild-type (WT) mice (24 months old), and in a murine model of Hutchinson-Gilford Progeria Syndrome, a human condition that results in premature aging. These mice carry the c.1827C>T; G609G mutation in the *Lamin A* gene, which causes aberrant splicing and accumulation of a truncated form of Lamin A called progerin (Osorio et al., 2011). We analyzed the small intestines as a control. In the small intestinal crypt, the number of cells expressing Ki67, a marker of proliferation, was reduced in aged and G609G mice compared with young WT mice (2 months old). In contrast, age did not affect the number of Ki67^+^ cells in the esophagus (Figures 1A,B). Similar results were obtained using another premature aging mouse model, PolG mice (Kujoth et al., 2005) (data not shown). We next analyzed senescence-associated beta-galactosidase (SA-βgal) activity, a canonical marker of senescence. Although SA-βgal activity was detected in the small intestines and kidneys of G609G mice, none was detected in the esophagus (Figure 1C). Moreover, age-associated induction of aging-related cyclin-dependent kinase inhibitors (p16 and p21), a p53-responsible, stress-inducible gene (GADD45b) and senescence associated secretory phenotype-related factors (Cxcl7, IL1β, and IL6) were not observed in the esophagus (Figure 1D). Notably, esophageal cells expressed pluripotency factors, regardless of the age of the mouse. Among these factors, SOX2 and NANOG proteins were quite specific to esophageal cells (Figures 1E,F), consistent with previous reports (Liu et al., 2013; Piazzolla et al., 2014). As reported, NANOG protein levels was lower in skin compared to those in the esophagus although mRNA levels seem differently regulated, which may reflect the complexity of post-transcriptional regulations of Nanog (Saunders et al., 2013; Piazzolla et al., 2014). Taken together, these results indicate that the esophageal epithelium expresses pluripotency factors and does not undergo aging-associated senescence *in vivo*.

### The Role of Myc in Self-Renewal of Esophageal Epithelial Cells *In Vitro*

We next derived primary mouse esophageal progenitor/basal cells (mEPCs) to analyze their ability to resist senescence in detail (Extended Data Supplemenary Figure S1). We optimized culture conditions based on previous reports (DeWard et al., 2014; Mou et al., 2016) and found that mEPCs could be homogeneously cultured on matrigel for > 50 passages (100 days) in SAGM medium that included A-83-01 (an ALK4/5/7 inhibitor), DMH-1 (an ALK2 inhibitor), CHIR99021 (a GSK-3β inhibitor), and Y-27632 (a ROCK inhibitor), hereafter referred to as ADCY. When cultured in ADCY, mEPCs propagated and expressed pluripotency factors. Withdrawal of ADCY resulted in rapid morphological changes and downregulation of markers of undifferentiation (Sox2, Nanog, and p63, a marker of basal epithelial cells), and telomere-related factors (mTert and mTerc), as well as the induction of Involucrin, a differentiation marker (Extended Data Supplementary Figure S2). These changes were seen in the presence or absence of Ca^2+^, which is known to induce keratinocyte differentiation, while a combination of Ca^2+^ addition and ADCY withdrawal synergistically induced Involucrin. We were able to derive mEPCs even from 24-month-old mice without any noticeable difference in derivation efficiency and cell morphology, compared to those derived from 2-month-old mice. This was not the case for skin keratinocytes and tongue epithelial cells, which were difficult to derive from aged mice (Extended Data Supplementary Figure S3). These results suggest that mEPCs do not exhibit replicative senescence. However, when challenged with Ras activation [*via* the retroviral overexpression of an activated form of Ras fused to the estrogen receptor (ER:Ras^V12^) and administration of the ER ligand, 4-hydroxytamoxifen (4OH)], mEPCs exhibited reduced levels of proliferation, larger cell size, loose cell-cell contacts, and a more differentiated state (Extended Data Supplementary Figures S4A–C). This observation implies that an oncogenic insult can trigger a senescence program, namely oncogene-induced senescence (OIS), which is in agreement with a previous report (Takaoka et al., 2004). The negative impact of Ras activation on cell proliferation was reversed by partial inhibition of the MAPK pathway, which was achieved by treating cells with of 0.01 μM PD0325901, a potent MEK inhibitor (Bain et al., 2007). Strong inhibition of the MAPK pathway (by treating cells with 1 μM PD0325901) dramatically impeded cell proliferation, suggesting that fine-tuning of MAPK, at least in part, contributes to EPC self-renewal (Extended Data Supplementary Figure S4D).

To determine whether pluripotency factors play an important role in mEPC self-renewal, we next manipulated the levels of Nanog, which was abundantly expressed in the esophagus and reported to have an oncogenic function in stratified epithelia (Piazzolla et al., 2014). RNAi-mediated knockdown of Nanog resulted in the poor propagation of mEPCs (Extended Data Supplementary Figure S5), as has been seen with Sox2 (DeWard et al., 2014). Thus, pluripotency factors are important for EPC self-renewal. Taken together, these results indicate that mEPCs express pluripotency factors and are deficient for replicative senescence, but are still capable of protecting themselves from tumor initiation *via* OIS.

**FIGURE 2:**
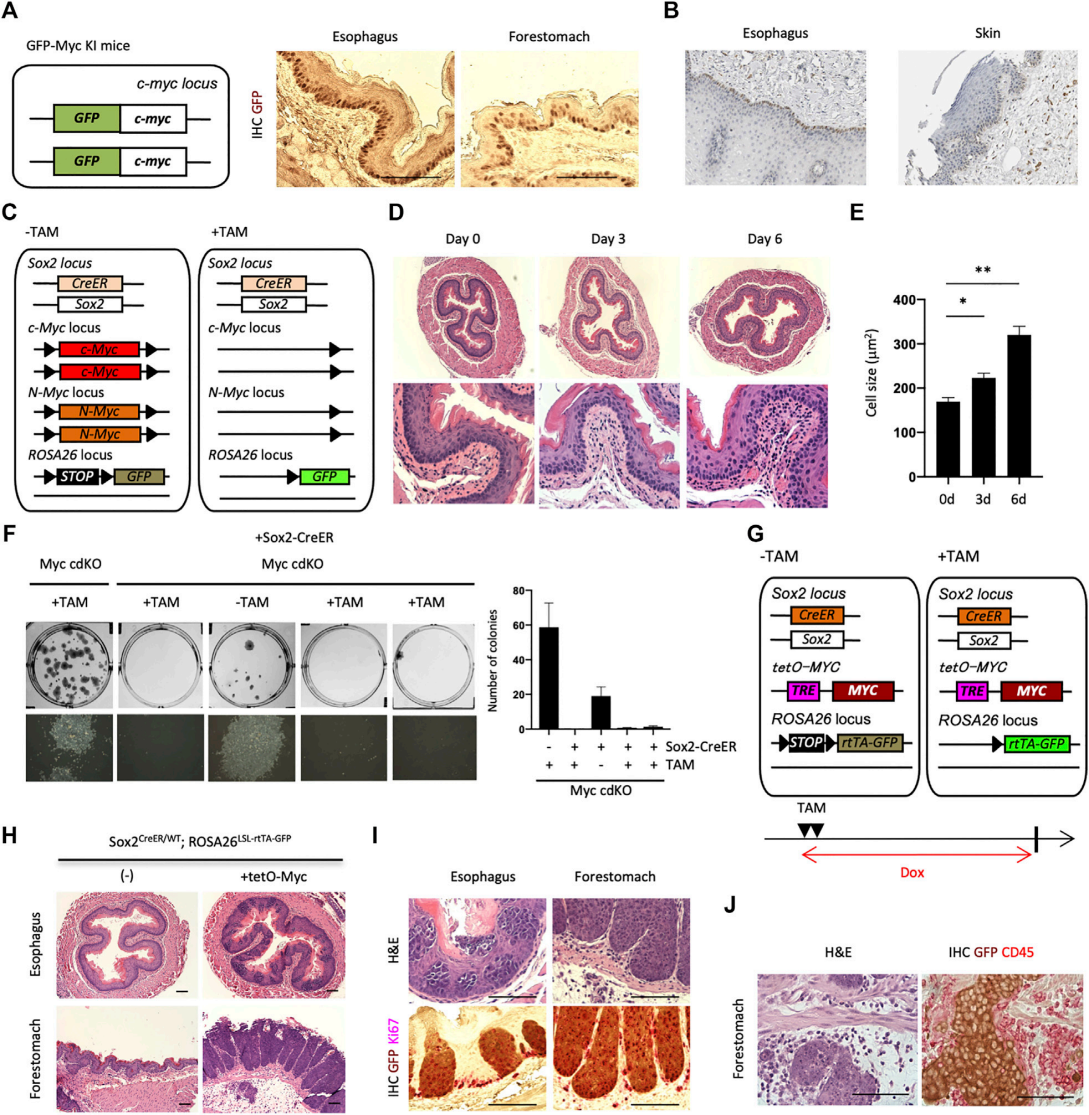
Loss-of-function and gain-of-function of Myc in the esophagus. **(A)** IHC for GFP on paraffin-embedded sections of the esophagus and forestomach from GFP-Myc Knock-in (KI) mice. Scale bars, 100 μm. Two mice were analyzed. **(B)** IHC image of MYC staining for the human esophagus and skin. MYC is largely expressed in esophageal basal layer. The data were kindly provided by the Protein Atlas Project publicly available (www.proteinatlas.org). **(C)** Schematic representation of Sox2^+^ cell-specific Myc conditional double knockout mice, *Sox2* ^CreER/WT^; *cMyc* ^Flox/Flox^; *nMyc* ^Flox/Flox^; *ROSA* ^*LSL-GFP/WT*^ (Myc cdKO). GFP can be used for lineage tracing purpose. **(D)** H&E staining for esophagi from Myc cdKO mice treated with TAM. Two mice were analyzed for each condition. **(E)** Cell size of esophageal basal cells. Two mice were used for each condition. Data represent the mean with SE. *p < 0.05, **p < 0.0001. **(F)** Clonogenic colony-forming assays. After cell isolation, 2,500 live cells were seeded per well in 6-well plate in ADCY medium. Twelve days after seeding, the cells were stained with Leishman’s stain to count the number of colonies. Each culture was derived from each indicated mice. Left, representative image of each condition. Right, quantification of colony number. Data represent the mean with SD (*n* = 3). **(G)** Schematic representation of *Sox2* ^CreER/WT^; *tetO-MYC*; *ROSA* ^LSL-rtTA-GFP/LSL-Luc^ mouse. TAM treatment eliminates STOP cassette in front of rtTA-IRES-GFP in *ROSA26* locus, which can activate transgenic *MYC* expression in a tetracycline- or doxycycline (Dox)-dependent manner. GFP expression allows for lineage tracing. The mice were collected 10 days after 2-days TAM treatment. Dox was administered to the mice in their drinking water (0.5 mg/ml). **(H,I)** H&E **(H)** and IHCs **(I)** on paraffin-embedded sections of the indicated tissues corrected from SMP^WT/WT^. Three mice were analyzed. Scale bars, 100 μm. **(J)** Inflammation along with tumor invasion in forestomach. Scale bars, 100 μm.

We next sought to understand the mechanism by which esophageal basal cells avoid replicative senescence. Using a Myc-GFP knock-in mouse model, we noticed that Myc is expressed in adult stem cells and progenitor cells associated with several tissues, including the esophagus and forestomach (Figure 2A, data not shown). Myc expression was relatively homogeneous in the basal layer of the esophageal epithelium, even though it is widely believed that Myc is transiently expressed during the G1 to S transition (Blackwood et al., 1991) and is relatively unstable. The MYC protein localizes to human esophageal basal cells at higher levels than seen in skin cells [according to the public data set from the Human Protein Atlas (Uhlen et al., 2015)] (Figure 2B). Myc is known to regulate self-renewal of pluripotent stem cells (PSCs) (Smith et al., 2010; Varlakhanova et al., 2010; Hishida et al., 2011). Notably, Myc depletion was reported to induce a pluripotent dormant state, indicating that Myc determines cell proliferation and growth arrest in PSCs. In addition, previous reports showed that c-Myc inactivation is associated with senescence in some cancer cells (Wu et al., 2007; Tabor et al., 2014; Alimova et al., 2019). Collectively, these results encouraged us to further investigate Myc’s function in supporting stemness.

To understand Myc’s role in inhibiting replicative senescence in mEPCs, we combined inducible Myc loss-of-function alleles [both c-Myc and N-Myc, as they are functionally redundant (Smith et al., 2010; Varlakhanova et al., 2010)] with Sox2-CreER (Figure 2C). We then treated Sox2^+^ cell-specific Myc conditional double knockout mice [*Sox2* ^CreER/WT^; *cMyc* ^Flox/Flox^; *nMyc* ^Flox/Flox^ (Myc cdKO)] with tamoxifen (TAM). Treated mice were dead within 7 days when TAM was administered continuously (data not shown). We therefore administered TAM for 3 days, collected esophagi, and performed H&E staining (Figure 2D). Esophageal basal cells were more sparsely larger in TAM-treated mice compared with untreated controls (Figure 2E and Extended data Supplementary Figure S6), which may reflect the loss of undifferentiated state as stated below. Further detailed analysis may provide deeper insight into contribution of Myc to differentiation and loss of stemness. To test whether Myc deletion affects self-renewal, we performed clonogenic colony-forming assays (Figure 2F). Only a few colonies were observed following Myc deletion, unlike that seen with controls, indicating that Myc is important for EPC self-renewal. We next examined whether Myc overexpression enhances mEPC proliferation, leading to tumors. To do so, we generated *Sox2* ^CreER/WT^; *tetO-MYC*; *ROSA* ^LSL-rtTA-GFP/LSL-Luc^ mice (Figure 2G) and treated them with TAM for 2 days and doxycycline (Dox, a tetracycline derivative) for 10 days, resulting in overexpression of *MYC* in Sox2^+^ cells. GFP was used to label *MYC* overexpressing cells in this mouse model. Ten days following Dox treatment, we observed the proliferation of GFP^+^ cells with abnormal morphologies in the esophagus and forestomach (Figures 2H,I). Invasive tumors were observed in the forestomach, with these invasive regions containing inflammatory cells, as assessed by H&E staining and localization of CD45, a marker of inflammatory cells (Figure 2J). An oncogenic role for Myc is also supported by the Oncoprint plot generated by cBioPortal (Cerami et al., 2012; Gao et al., 2013) (https://www.cbioportal.org/) from the Cancer Genome Atlas (TCGA), which indicates that MYC is frequently amplified (27%) in esophageal cancers (Extended data Supplementary Figure S7). Taken together, these results indicate that Myc is required for mEPC self-renewal while overexpression of Myc results in tumor formation.

### The Role of Myc in Self-Renewal of Esophageal Epithelial Cells *In Vitro*

**FIGURE 3:**
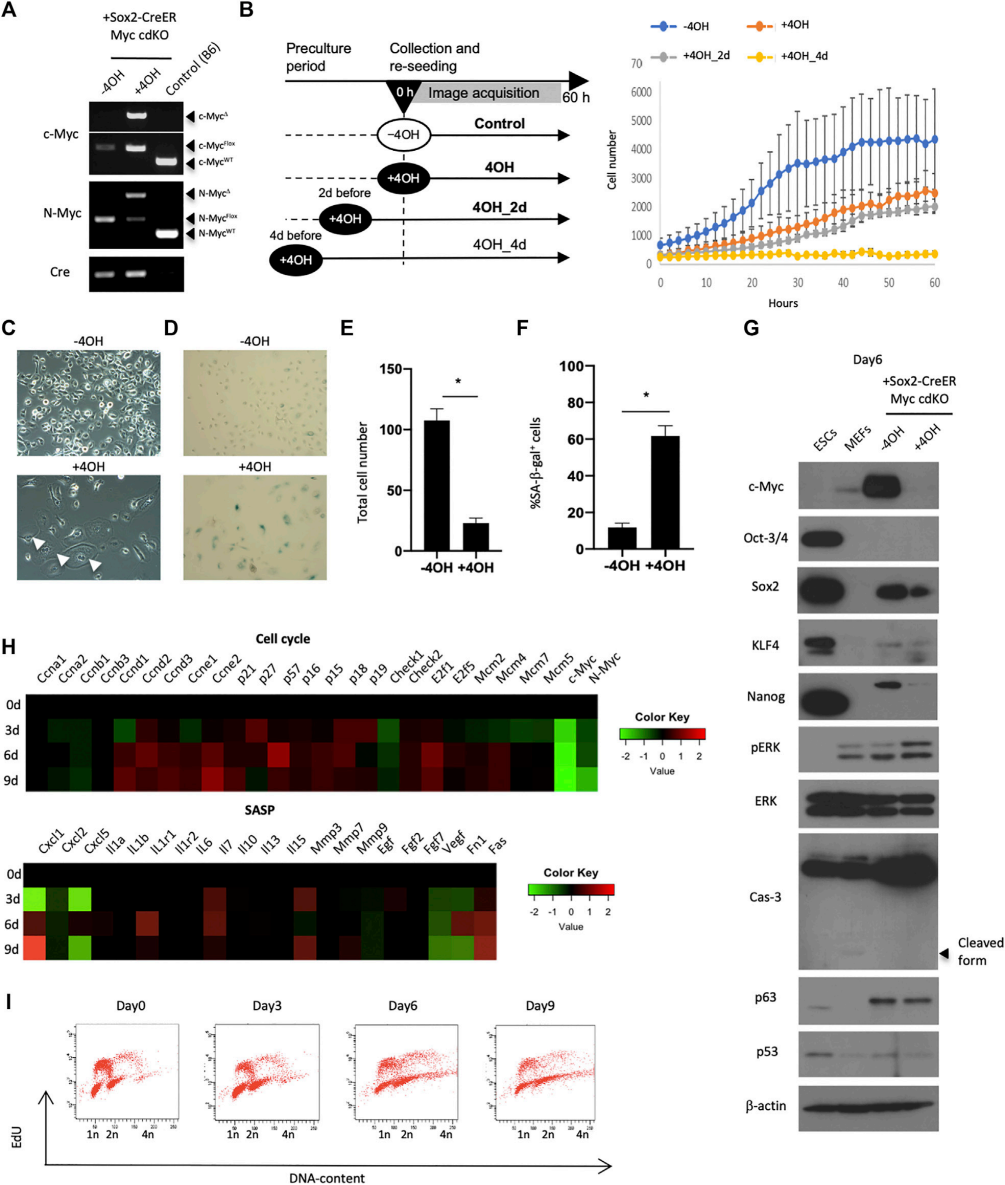
Requirement of Myc for preserving self-renewal of esophageal epithelial cells. **(A)** Genotyping to confirm Myc knockout. The cells were treated with 0.1 μM 4OH for 3 days and lysed for genomic DNA purification. PCR reactions were performed using purified genomic DNA for WT, Flox and deleted (Δ) alleles of c-myc and N-Myc. **(B)** Live-imaging of Myc cdKO mEPCs. Left, image acquisition scheme. Right, image-based cell count. **(C)** Image of untreated and the Myc cdKO mEPCs treated with 4OH for 9 days. White arrow indicates multinuclear cells. **(D)** SA-βgal staining in Myc cdKO mEPCs treated with 4OH for 9 days. Data represent the mean with SE (*n* = 6). *p < 0.0001. **(E)** Quantification of total cell number in **(D)**. Data represent the mean with SE (*n* = 6). *p < 0.0001. **(F)** Quantification of SA-βgal-positive cells in **(D)**. Data represent the mean with SE (*n* = 6). *p < 0.0001. **(G)** Western blotting for Myc cdKO mEPCs treated with 4OH for 6 days. **(H)** Nanostring-based gene expression analysis. Myc cdKO mEPCs were treated with 4OH and samples were collected at the indicated time-points. RNAs were isolated and subjected to Nanostring RNA detection. **(I)** Cell cycle analysis of Myc cdKO mEPCs by FACS.

We next sought to understand Myc’s role in EPC self-renewal in more detail by deriving mEPCs from Myc cdKO mice. We confirmed that 4OH treatment induced recombination at both the *c-myc* and *N-myc* loci, effectively knocking out both *Myc* genes (Figures 3A,E,F). Live-imaging experiments using the IncuCyte system revealed that 4OH-treated cells showed less proliferation (Figure 3B). As observed *in vivo*, enlarged cells (>1,000 μm^2^) also appeared and became more prevalent after several rounds of cell division (Figures 3C,E and Extended data Supplementary Figure S8). Enlarged cells were largely positive for SA-βgal staining (Figures 3D,F) and most of them could not undergo cell division (Extended Data Supplementary Figure S9). Western blotting revealed decreased levels of pluripotency factors, such as Sox2 and Nanog, whereas p63 levels were not affected (Figure 3G). We did not detect an accumulation of p53 protein, nor an increase in Caspase-3 cleavage, implying that apoptosis was not induced by Myc deletion. Rather, cyclin-dependent kinase inhibitors, as well as Cyclin D and E, were upregulated, whereas Cyclin A and B were downregulated (Figure 3H), as assessed by gene expression profiling using Nanostring technology. These analyses also showed that Myc deletion affected genes associated with MAPK and PI3K (Extended Data Supplementary Figure S10). In agreement with the dysregulation of cell cycle-associated genes, cell cycle analysis revealed fewer cells in S-phase and more endoreplication (Figure 3G), which is consistent with the emergence of multinuclear cells (Figure 3C). These multinuclear cells were not able to undergo cell division, as revealed by live-imaging (data not shown). Endoreplication with an enlarged morphology is typical of differentiated keratinocytes (Gandarillas et al., 2000). Multinuclear enlarged cells were also found in the absence of 4OH treatment, albeit in small numbers, and may therefore reflect spontaneous differentiation. Thus, the emergence of these cells cannot be attributed to Myc knockout, but may result from loss of an undifferentiated state after 4OH treatment. The dependence on Myc for self-renewal was also observed in human esophageal epithelial cell lines (EPC1 and EPC2) (Extended Data Supplementary Figure S11). Taken together, these results indicate that Myc is required for EPC self-renewal associated with the resistance to senescence.

### The Role of Mitochondria in Esophageal Cells on Suppressing Cellular Senescence

**FIGURE 4:**
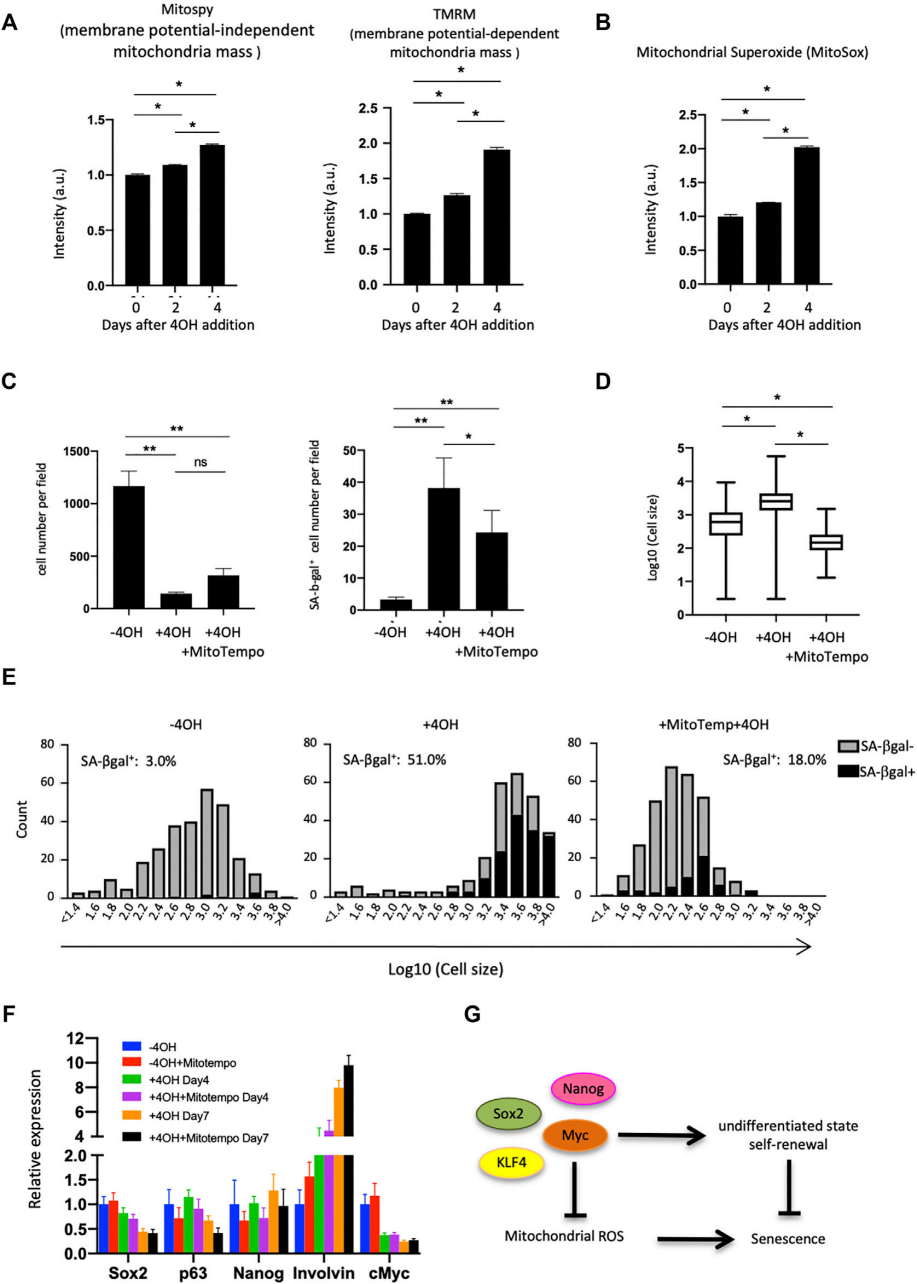
Mitochondrial analysis in Myc cdKO mEPCs. **(A)** Mitochondrial quantity and activity in Myc cdKO mEPCs treated for 2 days. Data represent the mean with SD (*n* = 3). *p < 0.0001. **(B)** Mitochondrial superoxide levels in 4OH-treaed cells. Data represent the mean with SD (*n* = 3). *p < 0.0001. **(C)** Rescue effect of Mitotempo. Left, representative image of each condition. Right, quantification of total cell number. Data represent the mean with SE. *ns* = non-significant, *p < 0.05, **p < 0.0001. **(D)** Rescue effect of Mitotempo on cell size. *p < 0.0001. **(E)** Histogram of the cell size distribution. **(F)** qPCR analysis for Mitotempo-rescued cells. Data represent the mean with SD (*n* = 3). **(G)** Proposed model of Myc function supporting self-renewal of the esophageal basal cells.

We next investigated the mitochondrial status within esophageal cells, as mitochondria play important roles in the induction of senescence (Gallage and Gil, 2016). Esophageal epithelial cells had lower membrane potential than skin epidermal epithelial cells (Extended Data Supplementary Figure S12), as assessed using TMRM, an indicator of membrane-potential-dependent mitochondria mass. This result encouraged us to analyze mitochondria in mEPCs. FACS analysis using MitoSpy and TMRM (for membrane potential-independent and -dependent mitochondria mass, respectively), showed that mEPCs had less mitochondrial membrane potential compared to Myc-deleted or Ca^2+^-treated cells (Figure 4A and Extended Data Supplementary Figure S13). Increases in membrane potential are known to produce reactive oxygen species and therefore we assessed mitochondrial superoxide levels following 4OH treatment. 4OH treatment increased mitochondrial superoxides, as assessed by MitoSox indicator (Figure 4B). Importantly, Mitotempo, a mitochondria-targeted antioxidant, reduced the number of SA-βgal positive cells, and resulted in smaller cells, while it did not affect total cell number (Figures 4C,D). Mitotempo did not affect loss of the undifferentiated state (Figure 4E), suggesting that an increase in mitochondrial membrane potential may be a consequence of the loss of an undifferentiated state, while helping to induce cellular senescence.

Senescence was thought to be a fundamental cellular process; however it has gradually been recognized that susceptibility to senescence is cell-type specific and indeed stem cells and progenitors are highly resistant to senescence. Our findings have revealed that esophageal epithelia are deficient for replicative senescence *in vivo*. Epithelial stem cells themselves are known to be resistant to aging; however skin epithelial stem cells do exhibit senescence in aged mice. Thus, esophageal epithelial cells may possess distinct mechanisms of self-renewal, which needs to be clarified. A previous report showed that human esophageal epithelial cells do not exhibit telomere shortening during aging, partly because of telomerase activity (Takubo et al., 1999). This supports our finding of the resistance to senescence in the esophageal epithelium. It is tempting to speculate that esophageal cells may have evolved characteristics of “perpetual youth” because they are turned over rapidly and must face damage and stress caused by continuous exposure to food and drink.

Of interest, Myc is homogeneously expressed in esophageal basal cells. This is a unique feature because Myc expression largely depends on the cell-cycle phase (Blackwood et al., 1991). Similar to MYC, Sox2 and Nanog are also expressed in esophageal epithelial cells, as reported (Liu et al., 2013; Piazzolla et al., 2014). These pluripotency factors might be key to sustaining the negligible senescence feature of esophageal epithelial cells. Indeed, Sox2 deletion lost self-renewing propensity (DeWard et al., 2014). It needs to be elucidated how MYC expression is homogenously sustained in basal cells of the esophageal epithelium.

Mechanistically, Myc inhibited senescence in the esophagus, and thereby functions as a double-edged sword, both inhibiting senescence and promoting tumorigenesis when overexpressed. This study is the first to show the physiological role of Myc (and Nanog) on preserving stemness in the esophageal epithelia by loss-of-function studies, presumably by working cooperatively with other pluripotency factors in this physiological context (Figure 4G), thus revealing a link between stemness and cellular aging.

## Data Availability

The original contributions presented in the study are included in the article/Supplementary Material, further inquiries can be directed to the corresponding author.
