# Supplementary figures and images for: Myc Supports Self-Renewal of Basal Cells in the Esophageal Epithelium
Source: Front Cell Dev Biol. 2022 Mar 4;10:786031. doi: 10.3389/fcell.2022.786031 (PMC8931341; doi:10.3389/fcell.2022.786031)

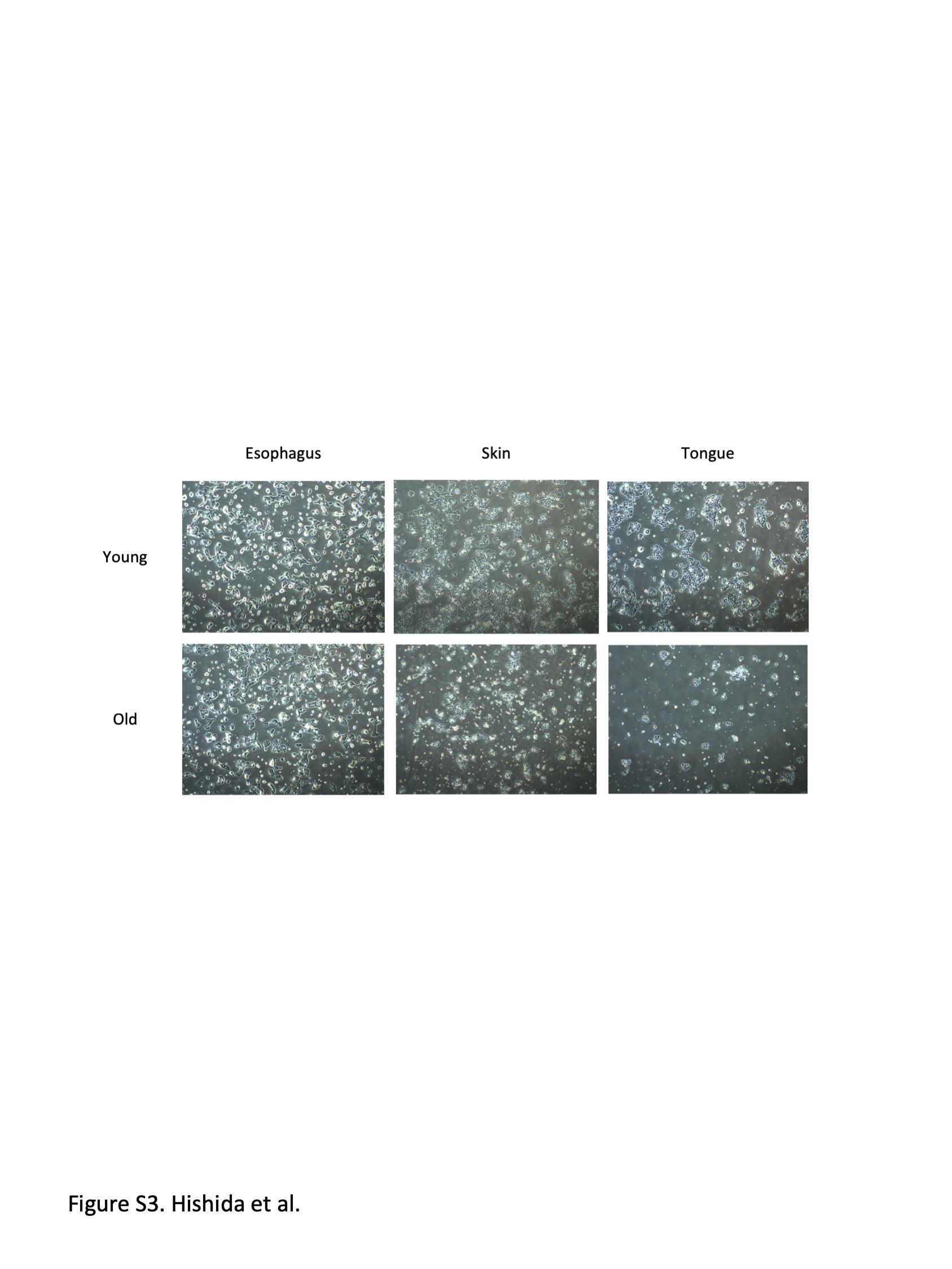

Supplement: Supplementary file 1 [file Image3.TIFF]

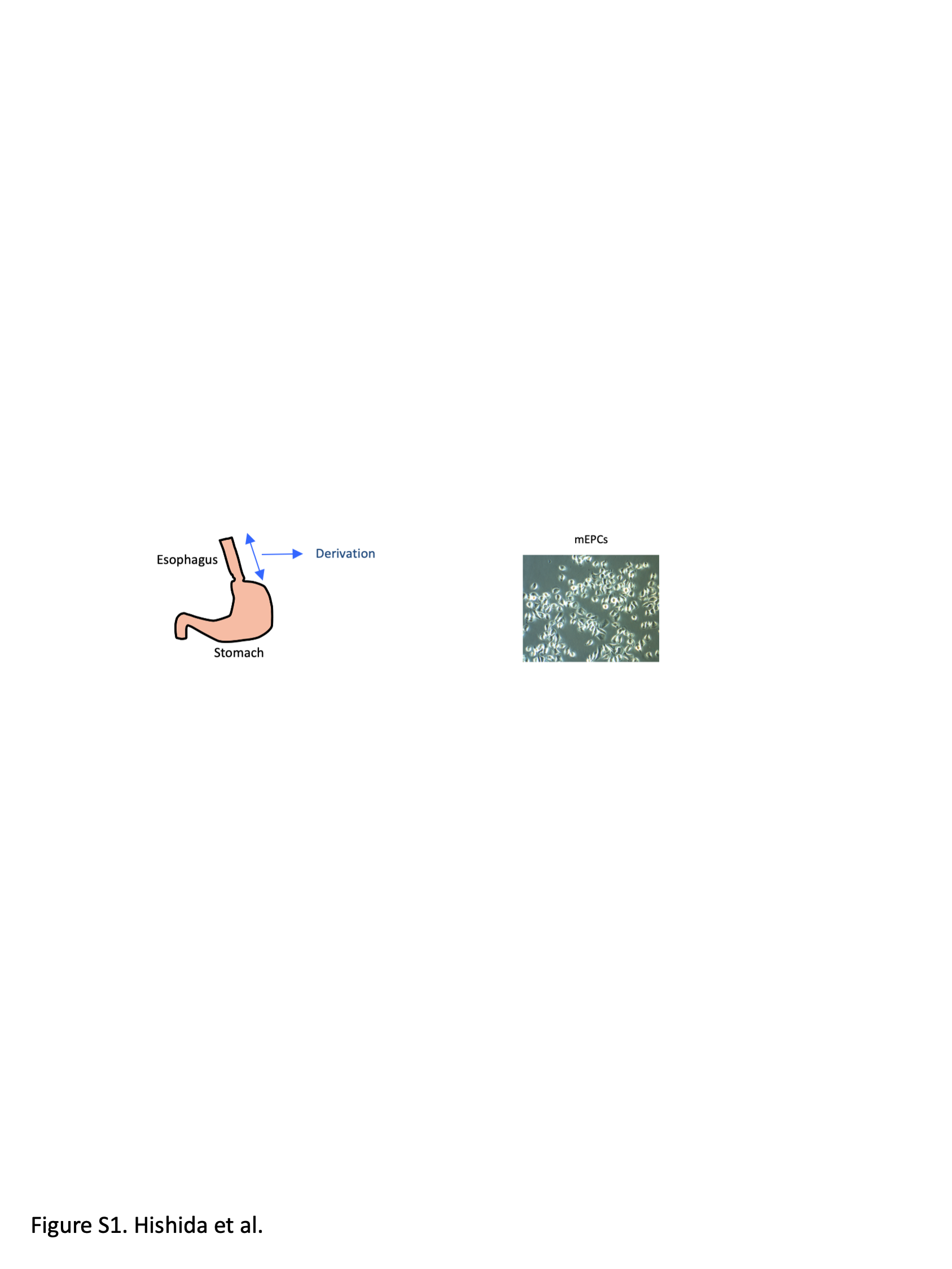

Supplement: Supplementary file 2 [file Image1.TIFF]

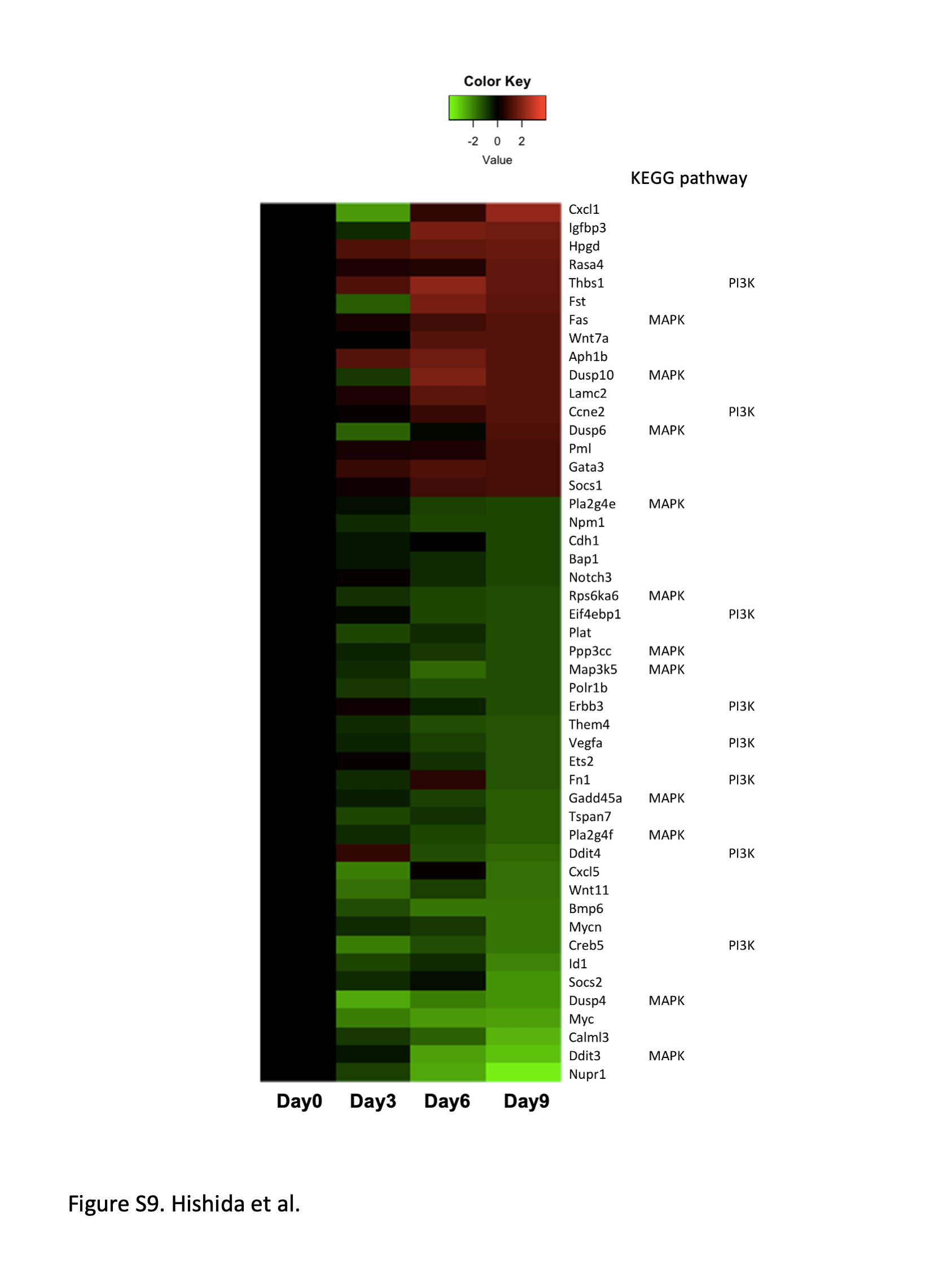

Supplement: Supplementary file 3 [file Image9.TIFF]

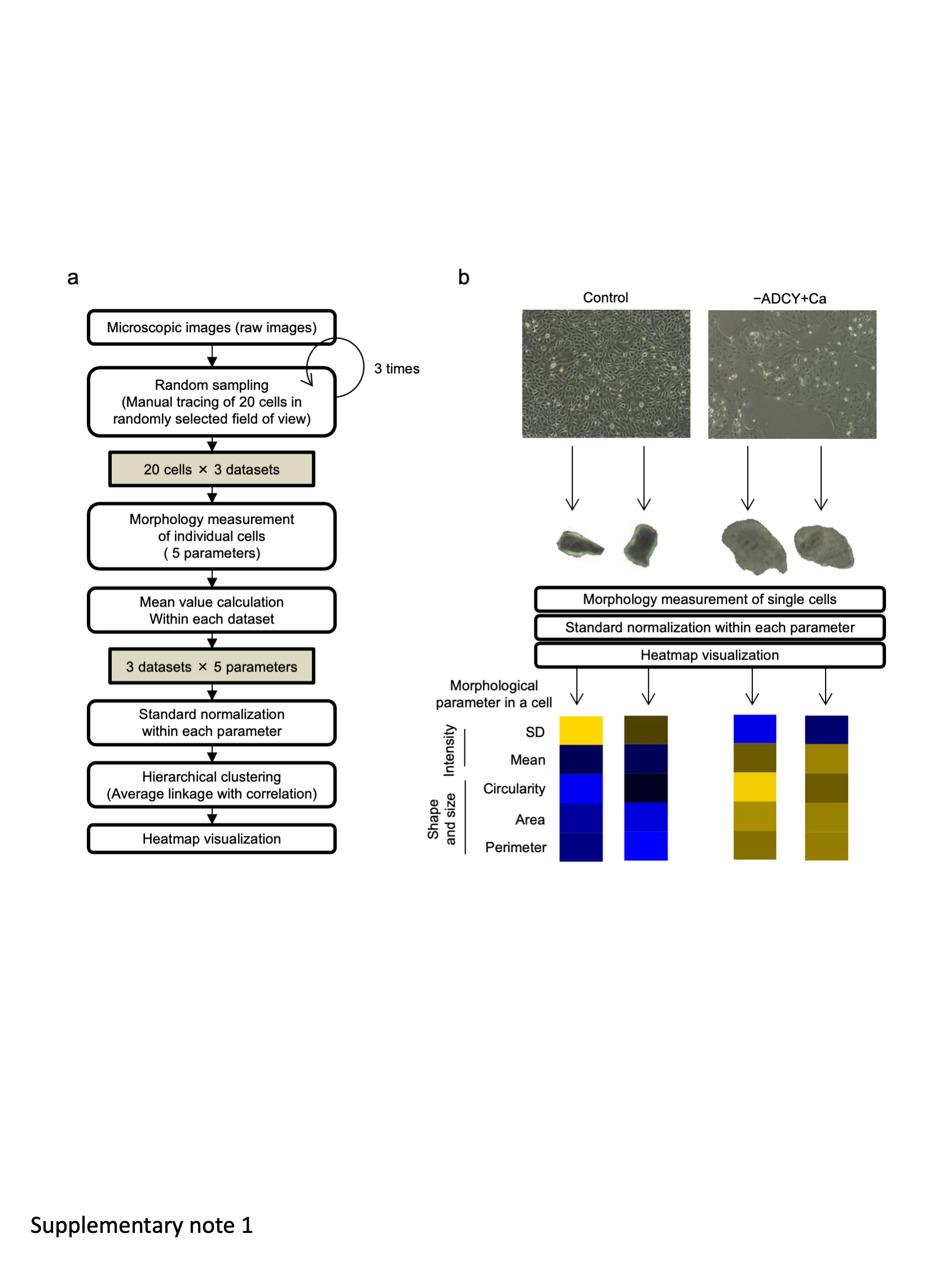

Supplement: Supplementary file 4 [file Image13.TIFF]

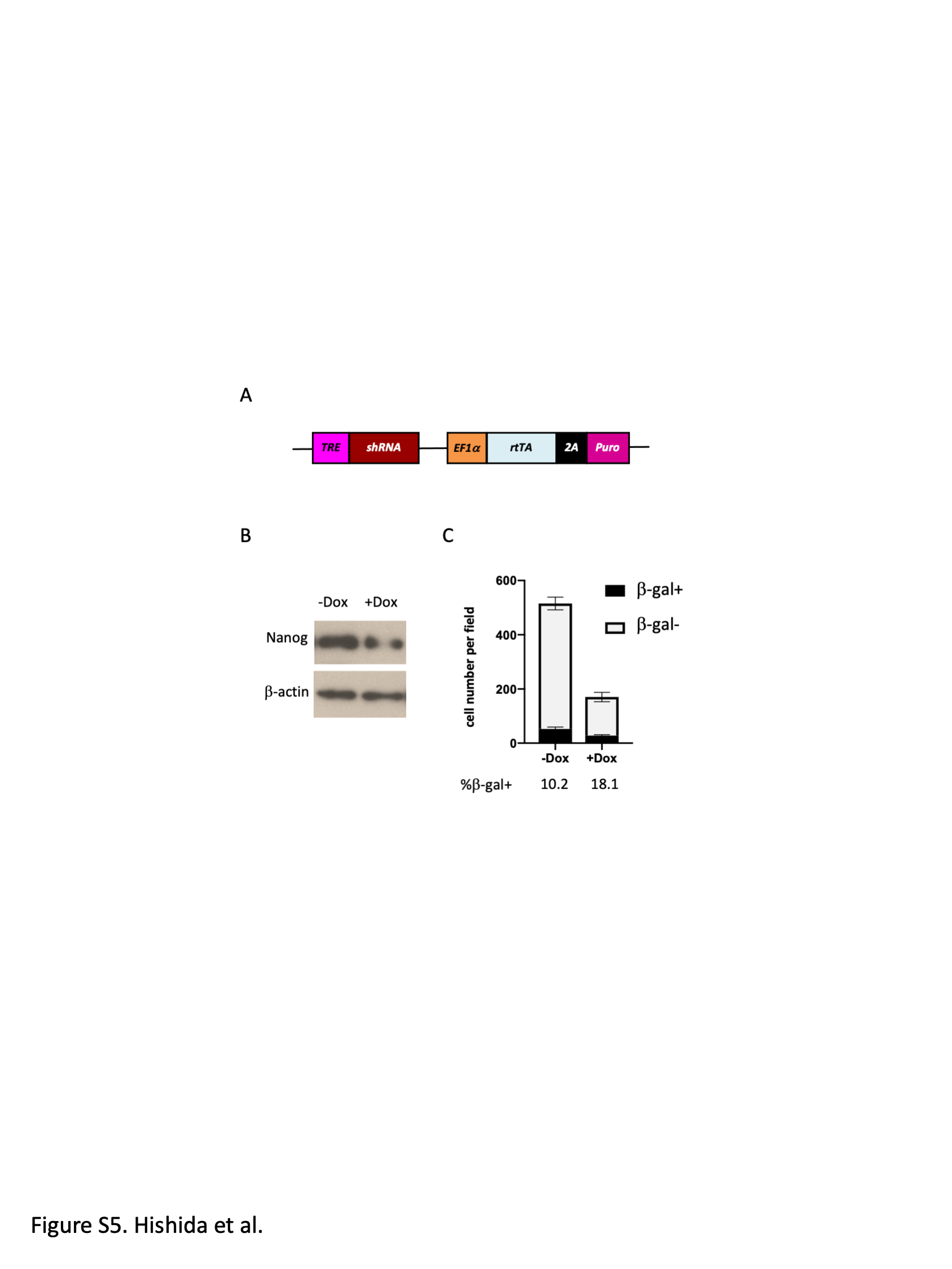

Supplement: Supplementary file 5 [file Image5.TIFF]

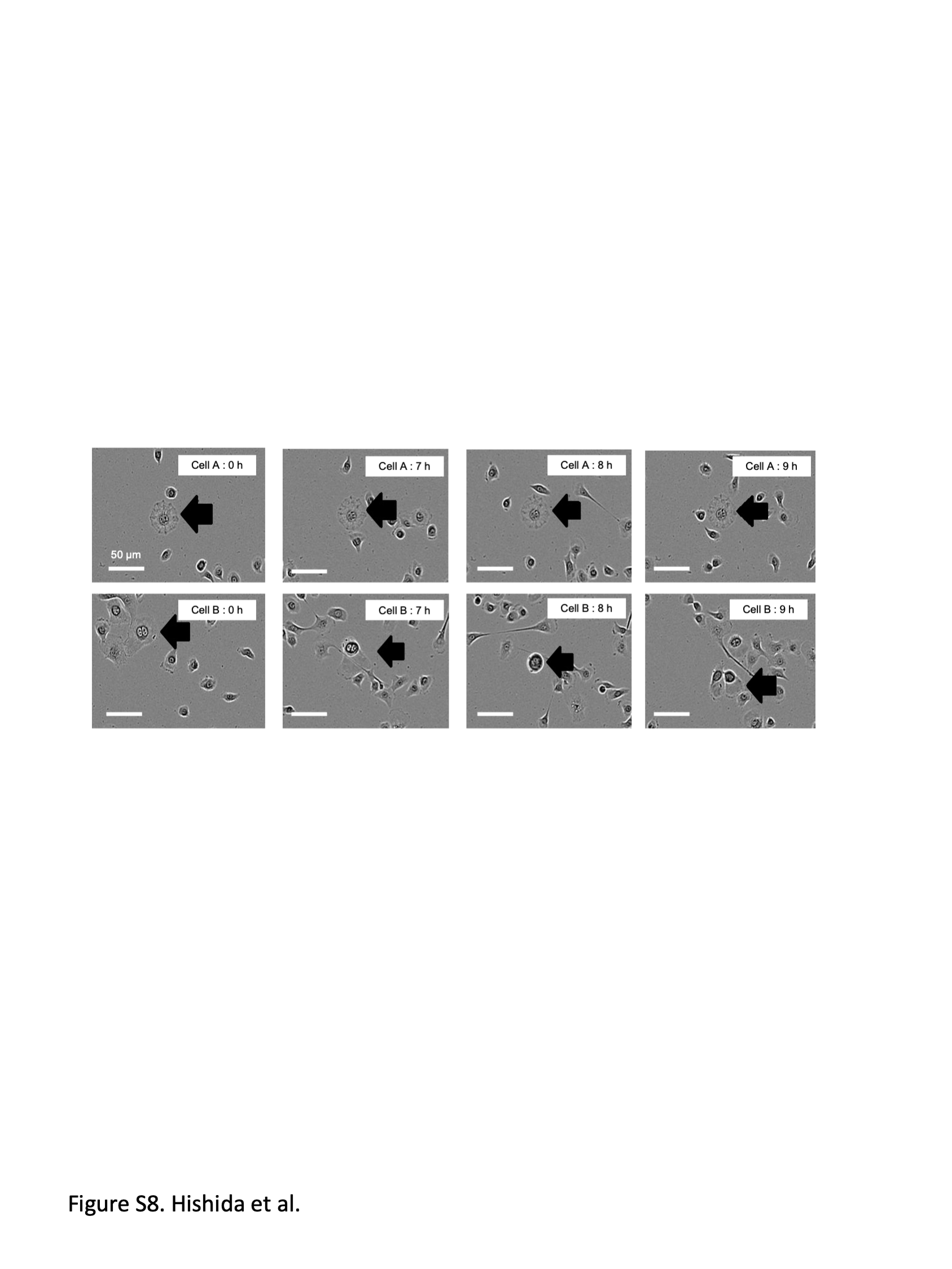

Supplement: Supplementary file 6 [file Image8.TIFF]

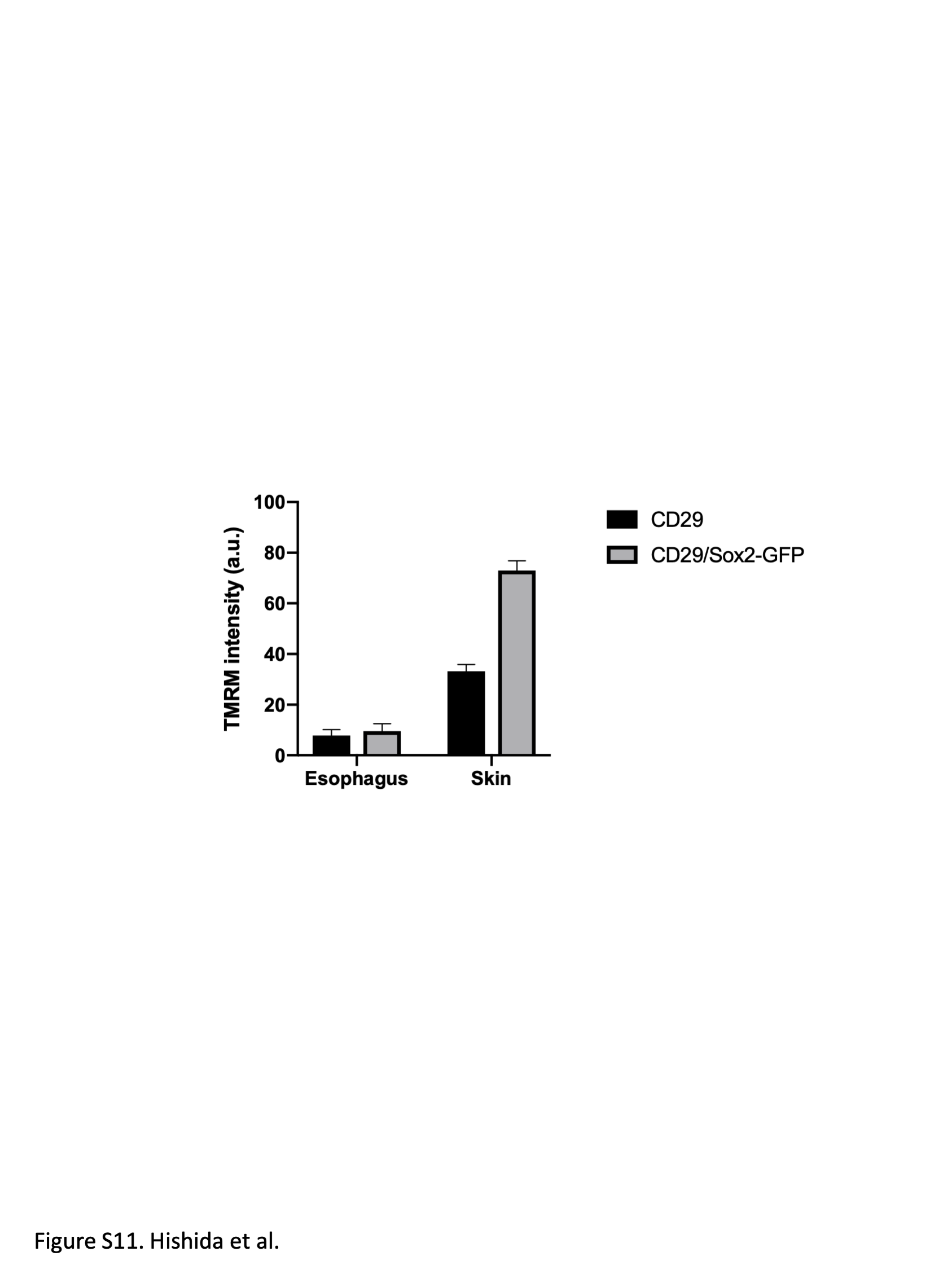

Supplement: Supplementary file 7 [file Image11.TIFF]

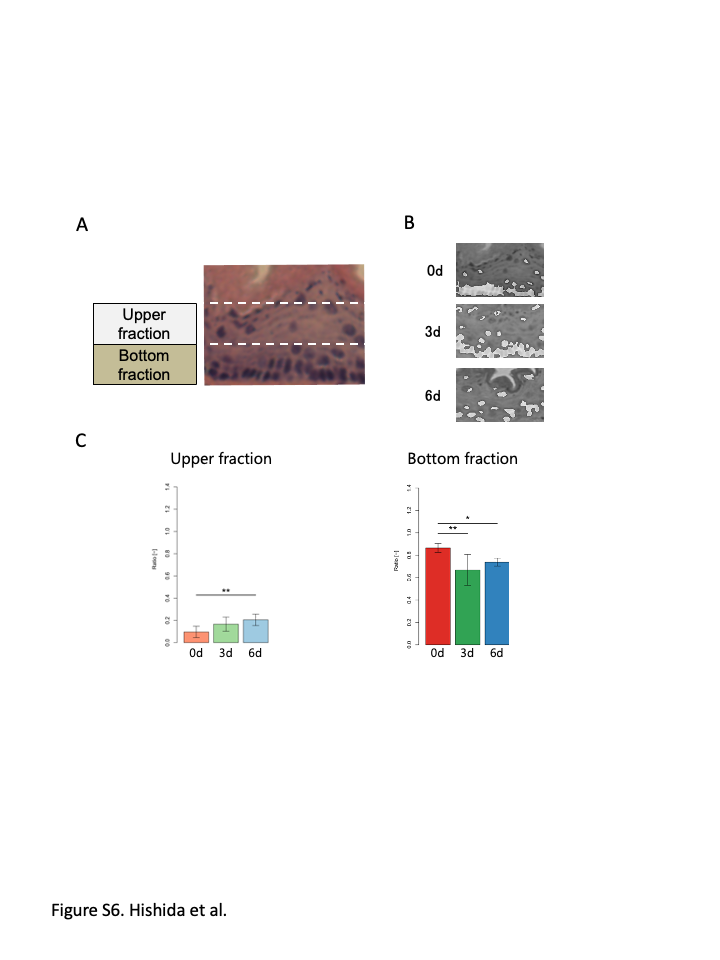

Supplement: Supplementary file 8 [file Image10.tiff]

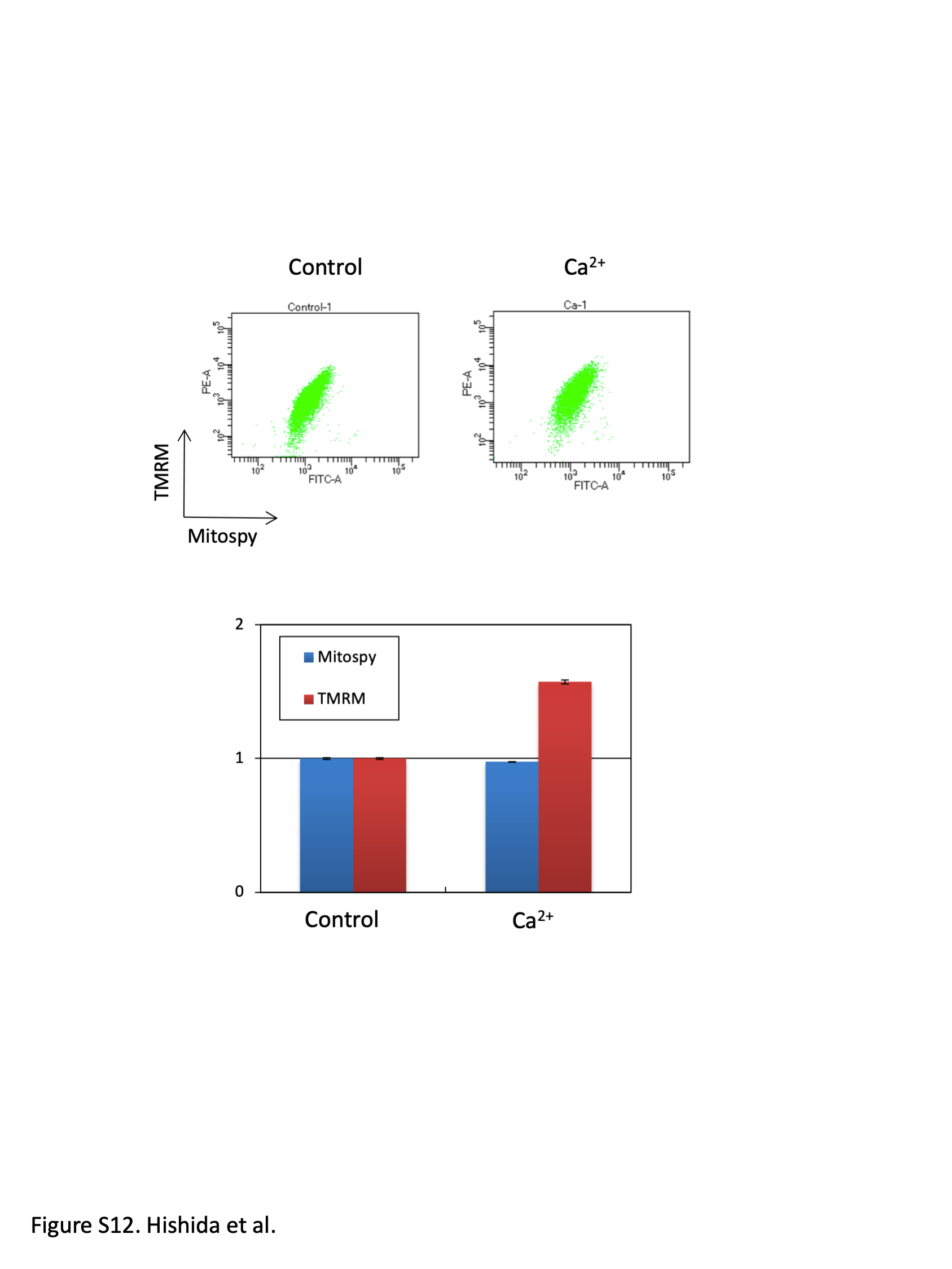

Supplement: Supplementary file 9 [file Image12.TIFF]

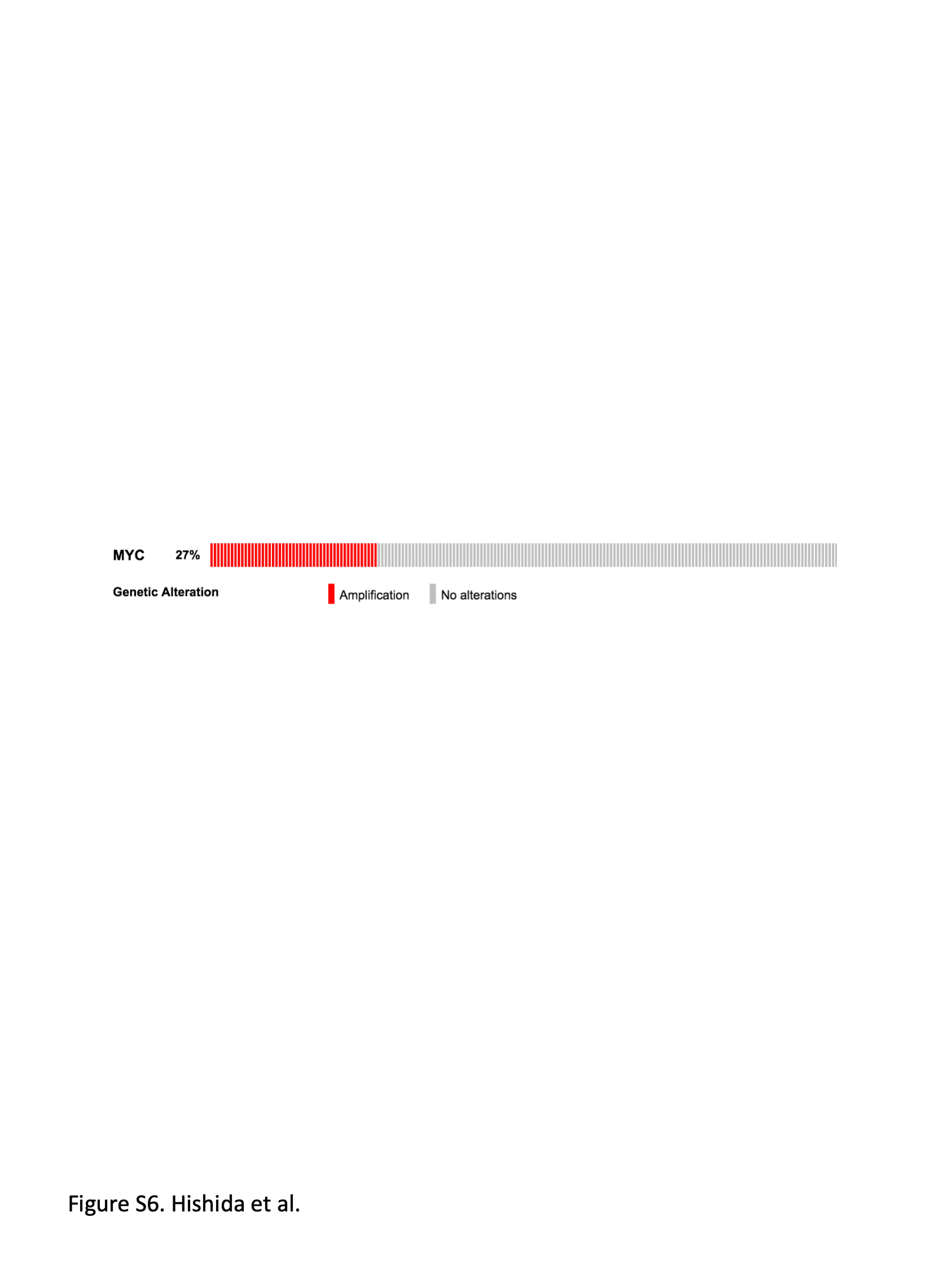

Supplement: Supplementary file 10 [file Image6.TIFF]

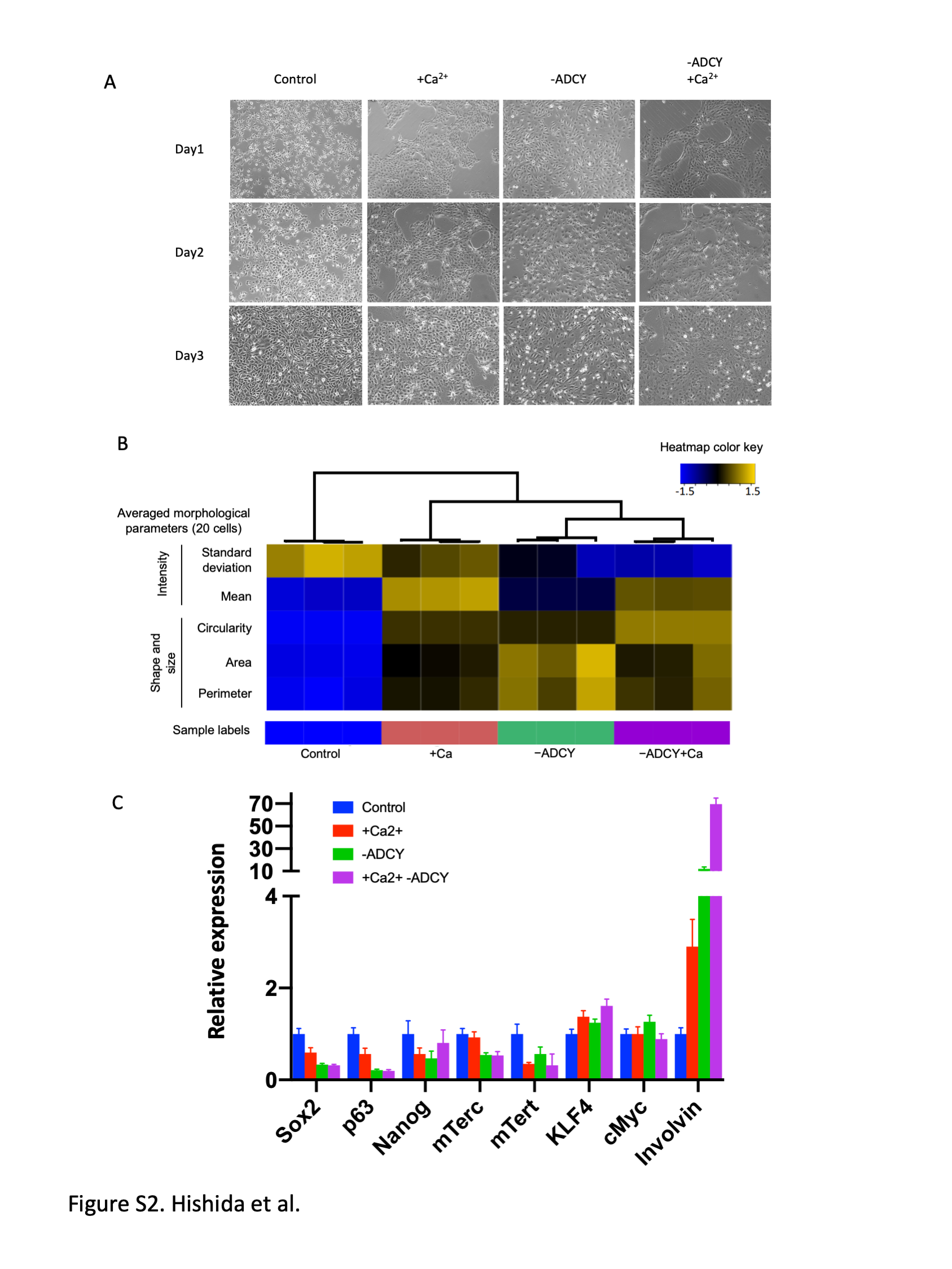

Supplement: Supplementary file 11 [file Image2.TIFF]

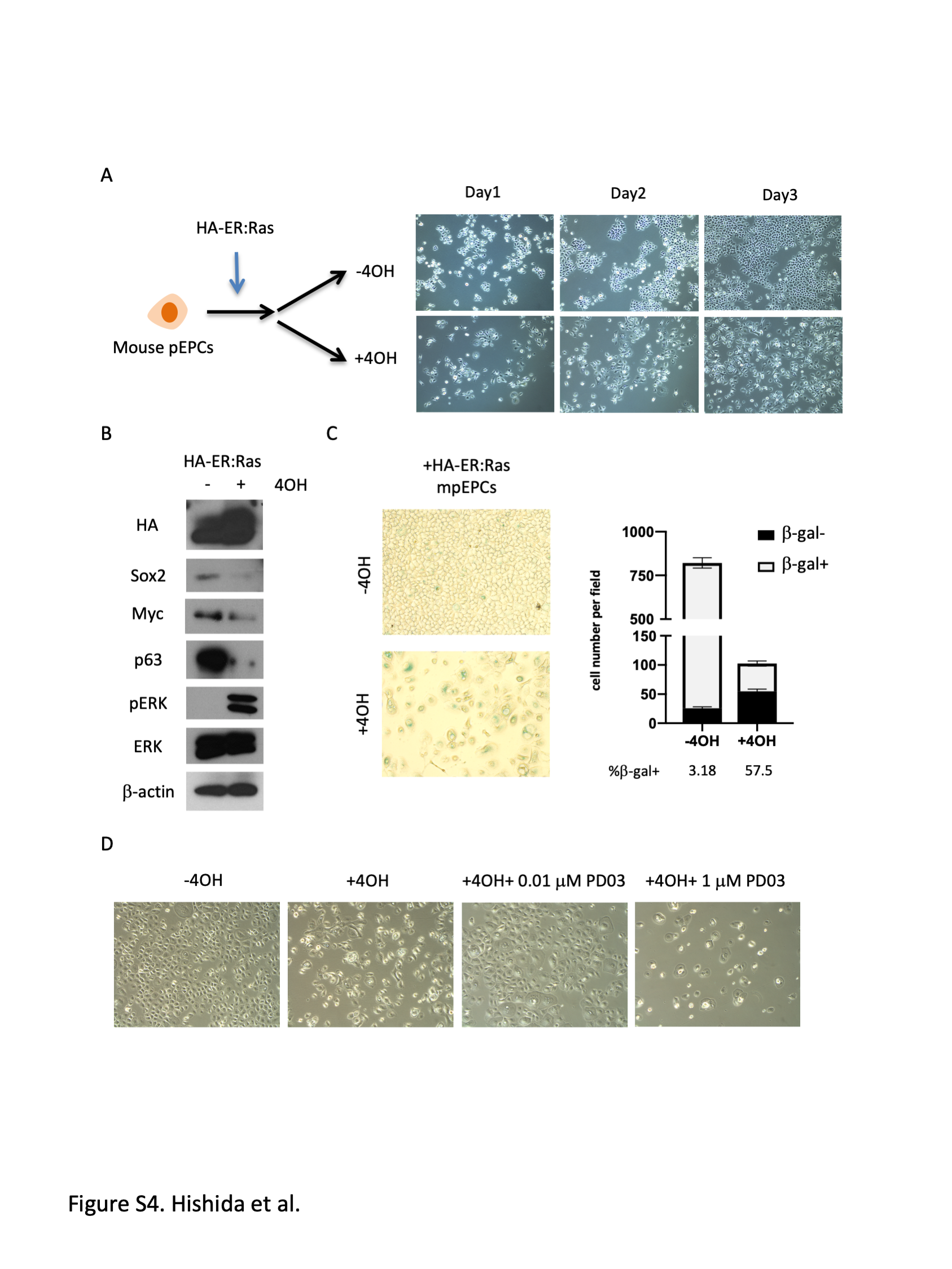

Supplement: Supplementary file 12 [file Image4.TIFF]

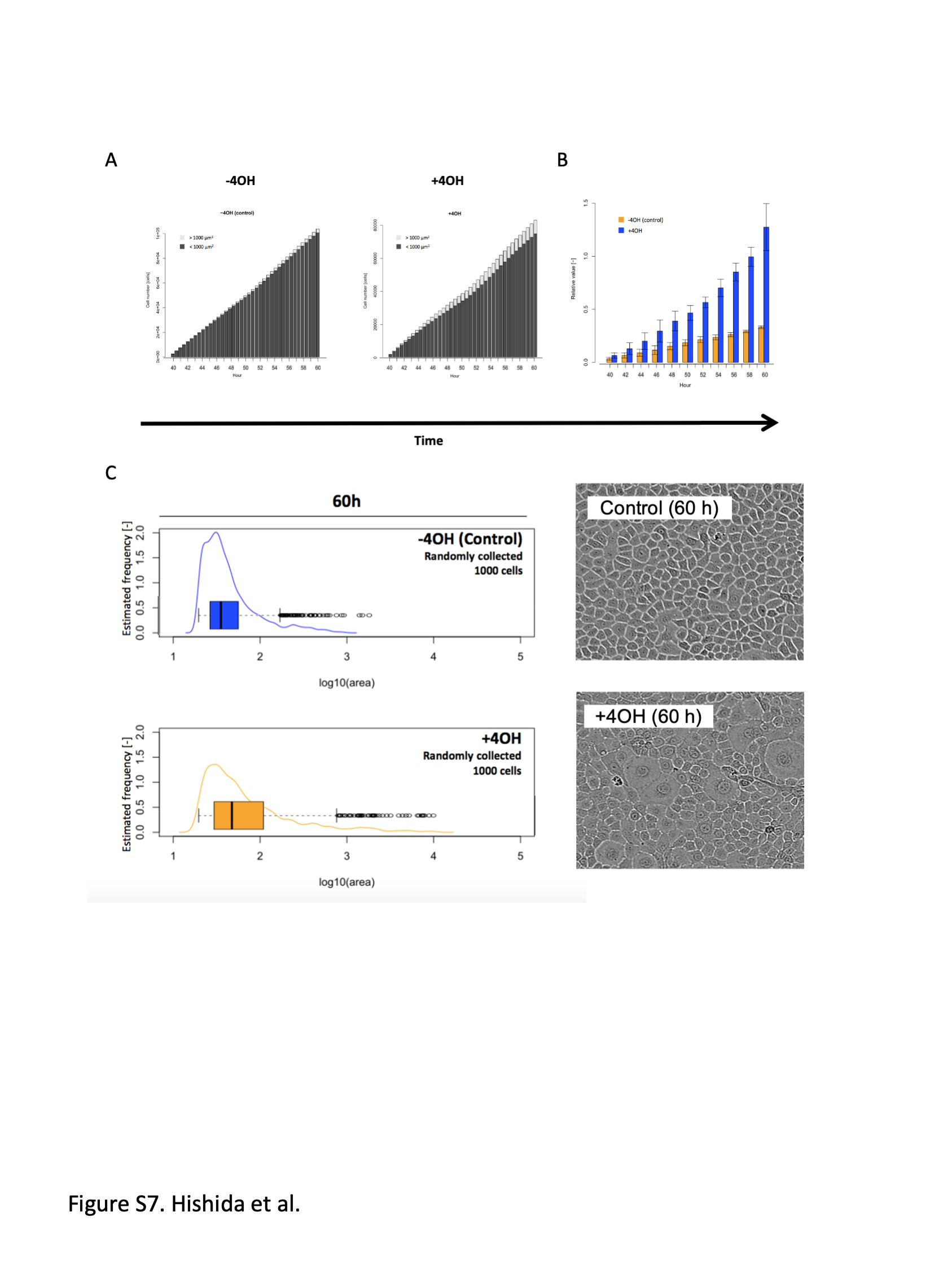

Supplement: Supplementary file 13 [file Image7.TIFF]
